# Highly efficient production of *Clostridium cellulolyticum* H10 d-psicose 3-epimerase in *Bacillus subtilis* and use of these cells to produce d-psicose

**DOI:** 10.1186/s12934-018-1037-1

**Published:** 2018-11-28

**Authors:** Lingqia Su, Fan Sun, Zhanzhi Liu, Kang Zhang, Jing Wu

**Affiliations:** 1 State Key Laboratory of Food Science and Technology, Jiangnan University, 1800 Lihu Avenue, Wuxi, 214122 China; 2 School of Biotechnology and Key Laboratory of Industrial Biotechnology Ministry of Education, Jiangnan University, 1800 Lihu Avenue, Wuxi, 214122 China; 3 International Joint Laboratory on Food Safety, Jiangnan University, 1800 Lihu Avenue, Wuxi, 214122 China

**Keywords:** d-Psicose 3-epimerase, *Bacillus subtilis*, High-cell-density fermentation, C/N ratio, d-Psicose

## Abstract

**Background:**

d-Psicose 3-epimerase (DPEase) catalyzes the isomerization of d-fructose to the rare sugar d-psicose, which may help prevent obesity, reduce blood sugar and blood fat, and inhibit intra-abdominal fat accumulation.

**Results:**

In this study, the DPEase of *Clostridium cellulolyticum* H10 was expressed in the food-grade host *Bacillus subtilis*. Optimization of the culture medium during shake-flask experiments yielded a DPEase activity of 314 U/mL. The optimal medium included 20 g/L peptone, 15 g/L corn steep powder, 5 g/L glycerol, and 1 mM Ca^2+^. Controlling the carbon source concentration was important because elevated concentrations can result in catabolite metabolic suppression (CCR). To avoid CCR and increase DPEase expression, we developed a fed-batch strategy in a 3.6-L fermenter. We altered the ratio of carbon source to nitrogen source (C/N) in the feeding medium and employed a constant feeding rate (6 g/L/h). This strategy improved the DPEase activity to 2246 U/mL (7.8 g/L), which is almost 15 times higher than that observed in the original shake-flask cultures. Finally, we used the DPEase-expressing *B. subtilis* cells to produce d-psicose from d-fructose, and a 28% conversion yield was achieved with these cells, demonstrating their potential use in d-psicose production.

**Conclusions:**

This is the first report to enhance recombinant DPEase production in *B. subtilis* using efficient and convenient fermentation strategy, and the DPEase yield is three times higher than the highest yield reported to date. The recombinant *B. subtilis* cells were further used in the efficient synthesis of d-psicose. This study provides a basis for the industrial production of d-psicose.

## Background

**Fig. 1:**
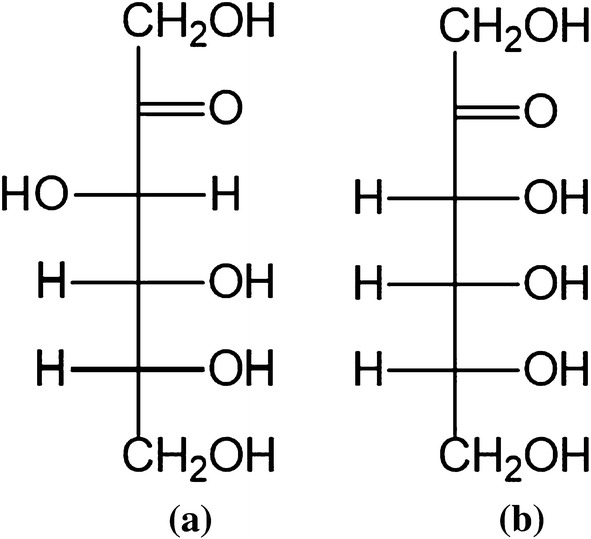
The molecular structures of d-fructose (**a**) and d-psicose (**b**)

The International Society of Rare Sugars first proposed the widespread application of rare sugars in human nutrition and health at its first academic conference in 2001. There are currently 34 species of rare sugar or sugar alcohol under consideration, including d-psicose, d-tagatose, d-allose, and xylitol. d-Psicose, an epimer of d-fructose at the C3 position, is 70% as sweet as sucrose, but its caloric value is only 0.3% of that of sucrose [1–3]. d-Psicose has recently attracted considerable attention from researchers because it has been linked with several useful activities, which include preventing obesity, reducing blood sugar and blood fat, and inhibiting intra-abdominal fat accumulation [4–6]. Because of its good physical and chemical properties, d-psicose has been used in food, dietary supplements, and pharmaceutical preparations, and has enormous market prospects [7].

d-Psicose is rarely found in nature, so most of the available material is produced by enzymatic synthesis. The conversion of d-fructose to d-psicose is catalyzed by d-psicose 3-epimerase (DPEase), which inverts the configuration of the hydroxyl group at the C3 position (Fig. 1). This reaction is reversible, and it usually achieves a maximum ratio of d-psicose:d-fructose between 20 and 33% [8]. There have been some recent reports of the production of rare sugars by whole-cell catalysis. For example, Zhang et al. produced d-psicose by using pretreated *Rhodobacter sphaeroides* cells [9] and Shin et al. increased the production of food-grade d-tagatose by pretreating and immobilizing *Corynebacterium glutamicum* cells [10].

DPEase is the key enzyme required to produce d-psicose from inexpensive d-fructose, therefore it is important to identify an active enzyme and improve its yield. DPEases from many sources have been discovered over the last few years, and most of them have been expressed as recombinant proteins in *Escherichia coli* [11–13]. These methods are useful for experimental purposes. However, industrial d-psicose production is meant to produce a food additive, so safety issues are particularly prominent. Due to the production of endotoxin, *E. coli* as a host presents a security problem and is not suitable for the expression of DPEase intended for use in the industrial production of d-psicose. *B. subtilis*, a food safety bacterium belonging to the group of microbes classified as generally regarded as safe (GRAS), widely used in the production of industrial enzymes due to absence of security issues [14, 15]. Therefore, the overproduction of heterologous proteins by high-cell-density fermentation in recombinant *B. subtilis* is highly valued. Since the culture medium and cultivation conditions may have substantial effects on protein production, it is important to determine the optimal conditions for the production of each particular protein in *B. subtilis*. During typical industrial fed-batch fermentations of *B. subtilis*, efficient protein expression often requires maintaining the carbon concentration at a low level in the medium. For example, Zhu et al. studied different glucose feeding approaches and step-wise dissolved oxygen control methods to improve *N*-acetylglucosamine production [16] and Shene et al. increased the recombinant enzyme concentration by feeding the carbon source slowly during fed-batch fermentation [17]. Huang et al. reported that high initial glucose concentrations cause product inhibition and used a dual exponential fed-batch strategy to prevent this inhibition [18]. However, this method is tedious and requires sophisticated control measures. Other components in culture medium might also have a significant effect on protein production. Chen et al. found that glucose and glycerol were not conducive to the expression of recombinant *B. subtilis* Rluc protein, and a high protein yield was obtained by reducing the amount of glucose and adding yeast extract in a 5-L fermenter [19]. Kwon et al. found that when the ratio of glucose to peptone in the feeding medium was 0.33:1, the activity of recombinant nattokinase reached 14,500 U/mL, which was 4.3 times higher than that in batch culture [20].

The past few years witnessed a number of reports describing the recombinant expression of DPEases in *B. subtilis* [21–23]. However, to the best of our knowledge, only a few studies have investigated DPEase expression levels in both *B. subtilis* and other hosts. In 2016, Sun and coworkers constructed a recombinant DPEase expression vector containing a xylose-inducible promoter that successfully expressed DPEase in *B. subtilis* [24]. Using this system, the expression level of DPEase reached 95 U/mL (2.6 g/L) in a 7.5-L fermenter with the fed-batch fermentation. To our knowledge, this was the highest production level prior to this study.

In the present study, a synthetic, codon-optimized gene encoding the DPEase from *Clostridium cellulolyticum* H10 was successfully expressed in *B. subtilis* strain CCTCC M 2016536 [25] using a vector containing the constitutive promoter P*amyE*. Initial experiments to optimize DPEase production were carried out in shake flasks. Further efforts to improve DPEase expression involved high-density culture carried out in a 3.6-L fermenter. The fed-batch fermentations were improved by optimizing the C:N ratio of the feeding solution. Finally, we studied the synthesis of d-psicose by whole-cell catalysis with varied wet cell dosage, temperature, and pH, respectively.

## Materials and methods

### Bacterial strain and vectors

The codon-optimized (based on *B. subtilis* preferences) gene encoding the DPEase from *Clostridium cellulolyticum* H10 was synthesized to Generay Co., Ltd (Shanghai, China). It was inserted in *B. subtilis*–*E. coli* shuttle vector pHY300PLK (Takara, Dalian, China) and expressed in *B. subtilis* CCTCC M 2016536 (constructed and stored by our laboratory).

### Shake-flask cultures

The Luria–Bertani (LB) medium contained (g/L) yeast extract 5.0, tryptone 10.0, and NaCl 10.0 was used during the production of seed cultures. The Terrific Broth (TB) medium contained (g/L) tryptone 12.0, yeast extract 24.0, glycerol 5.0, K_2_PHO_4_ 12.5, and KH_2_PO_4_ 2.3 was employed for protein expression. Seed cultures were initiated by inoculating 10 mL of LB medium supplemented with 20 mg/L tetracycline in a 50-mL shake flask with 20 μL of glycerol stock strain. These seed cultures were cultivated at 37 °C in a rotary shaker at 200 rpm for 8 h. Then, 2.5 mL of the seed culture was transferred to 50 mL of TB medium supplemented with 20 mg/L tetracycline in 250-mL shake flask. This culture was shaken at 200 rpm for 48 h at 33 °C.

### Media, feeding solutions and cultivation in 3.6-L fermenter

Seed cultures were prepared in LB medium as described in the previous section. A modified semisynthetic medium was used for cultivation in a 3.6-L fermenter. The initial batch medium contained (g/L) peptone 20.0, corn steep powder 15.0, glycerol 5.0, K_2_PHO_4_ 12.5, KH_2_PO_4_ 2.3, (NH_4_)_2_-H-citrate 1.0, (NH_4_)_2_SO_4_ 2.7, MgSO_4_·7H_2_O 1.0, and CaCl_2_ 0.2, as well as 3 mL/L trace metal solution [26]. The feeding solution contained 500.0 g/L total glycerol and nitrogen (industrial grade), 7.9 g/L MgSO_4_·7H_2_O 7.9, and 20 mL/L trace metal solution.

The initial batch medium for fed-batch cultivation (0.9 L) in a 3.6-L fermenter (Labfors 5, Infors-HT Co., Ltd) kept at 33 °C and pH 7.0 was supplemented with 20 mg/L tetracycline and then inoculated with a 100-mL seed culture. The pH was controlled with 20% phosphoric acid and ammonium hydroxide. When the glycerol present in the initial medium was consumed, as indicated by a sudden increase in the dissolved oxygen and a decrease in the agitation rate, we began adding the constant feeding solution to the culture. To maintain the dissolved oxygen level at about 30% of air saturation during the fermentation process, the agitation speed was cascaded between 200 and 700 rpm.

### Determination of biomass

Cell growth was monitored by measuring the optical density of the culture broth at 600 nm (OD_600_) using a spectrophotometer (BioPhotometer plus, Eppendorf Co., Hamburg, Germany). To determine the dry cell weight (DCW), 5 mL of culture broth was centrifuged at 12,000*g* for 10 min. The pelleted cells were washed twice with 0.9% (w/v) NaCl and centrifuged at 12,000*g* for 10 min, then dried to constant weight at 105 °C.

### Preparation of DPEase and SDS-PAGE

A sample of the culture broth was diluted to an OD_600_ of around 5 and centrifuged at 12,000*g* for 10 min. The pelleted cells were resuspended in 1 mL HEPES buffer (20 mM, pH 8.0), then the cells were incubated with 30 μL lysozyme (20 mg/mL) at 37 °C for 30 min before disruption by sonication for 10 min (pulse on for 2 s and pulse off for 3 s). The cell debris was removed by centrifugation at 12,000*g* for 5 min.

The protein content of the culture broth was assessed using sodium dodecyl sulfate polyacrylamide gel electrophoresis (SDS-PAGE) analysis. Protein bands were stained with Coomassie Brilliant Blue R-250 dye according to standard procedures.

### DPEase activity assay

DPEase activity was measured via determining the quantity of produced d-psicose. The reaction mixture (1 mL final volume) contained d-fructose (80 g/L), HEPES buffer (20 mM, pH 8.0) and Co^2+^ (0.1 mM), and the reaction was initiated by the addition of enzyme solution (200 μL). The reaction was incubated at 55 °C for 10 min, and then stopped by placing the tube in boiling water for 10 min. The amount of generated d-psicose was determined by using a high-performance liquid chromatography (HPLC) method. The HPLC apparatus was equipped with a Shodex™ Asahipak™ NH2P-50 4E column and a refractive index detector (Agilent Technologies, Palo Alto, CA, USA). The column was maintained at 35 °C and eluted with 75% acetonitrile at a flow rate of 0.8 mL/min. One unit of DPEase activity was defined as the amount of enzyme that catalyzed the production of 1 μmol of d-psicose per minute.

## Results and discussion

### Recombinant DPEase expression in *B. subtilis*

**Fig. 2:**
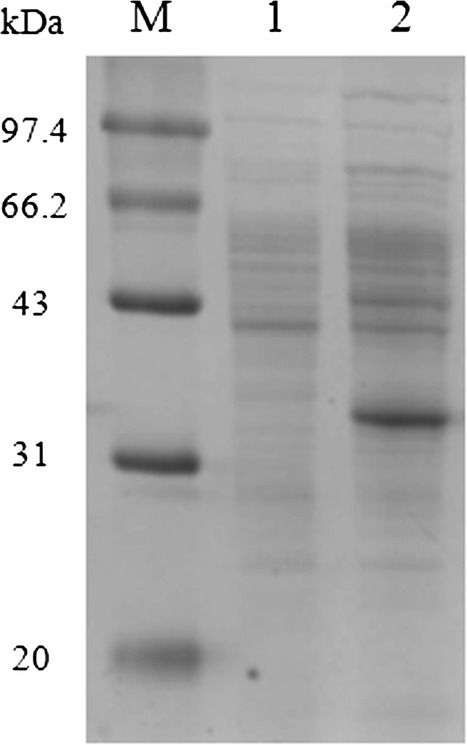
SDS-PAGE analysis of the recombinant DPEase from *B. subtilis*. Lane M, protein markers; Lane 1, intracellular proteins from *B. subtilis* containing the expression plasmid (pHY300PLK) lacking the DPEase-encoding gene (negative control); Lane 2, intracellular proteins from *B. subtilis* containing the expression plasmid pHY300PLK-DPEase

In this study, a DPEase expression plasmid for use in *B. subtilis* was constructed by inserting a synthetic, codon-optimized DPEase gene into the plasmid vector pHY300PLK. The 882 bp insert encodes a 294 amino-acid protein with a molecular weight of 33 kDa. In the initial experiment, recombinant DPEase expression was assessed in TB medium at 33 °C in a shake flask. As shown in Fig. 2, SDS-PAGE analysis demonstrated that the DPEase from *C. cellulolyticum* H10 was successfully expressed in *B. subtilis.* The highest DPEase activity (148.9 ± 3.0 U/mL) was reached at 48 h of cultivation. As low yield is a bottleneck to obtain sufficient DPEase for industrial applications, we sought to enhance DPEase production.

### Effects of process conditions on the production of DPEase in shake flasks

#### Nitrogen sources and nitrogen source concentrations

**Fig. 3:**
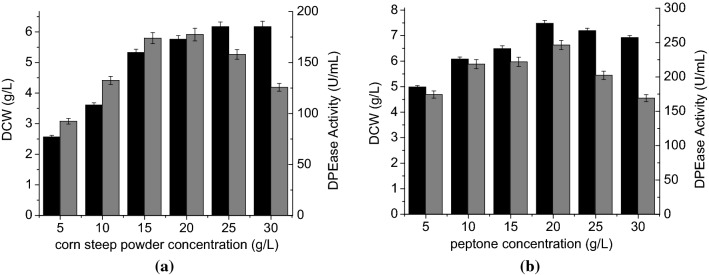
Effects of complex nitrogen sources on cell growth and DPEase activity. **a** Corn steep powder; **b** peptone. DCW (black), DPEase activity (gray)

It is well known that the composition of the culture medium is critical to protein expression. Readily available, inexpensive materials are normally employed in industrial scale production. TB medium, which is commonly used for cell growth and fermentation, contains an abundance of nutrients. To select a suitable nitrogen source for use with the TB medium, we replaced the nitrogen-containing components of TB medium with different nitrogen sources and assessed the effects of their concentrations on DPEase production. As shown in Table 1, the nitrogen-containing components of TB medium were replaced by yeast extract, peptone, corn steep powder, yeast decoction, and cottonseed meal, respectively. The maximum biomass (6.2 ± 0.2 g/L) and highest DPEase activity (213.7 ± 4.8 U/mL) were obtained with cottonseed meal at a concentration of 35 g/L. Unfortunately, cottonseed meal is inappropriate for most industrial applications at this high concentration due to poor solubility. Since peptone at 25 g/L and corn steep powder at 15 g/L yielded DPEase activities (189.9 ± 2.8 and 167.0 ± 3.3 U/mL, respectively) just lower than that observed with cottonseed meal, but at a lower concentration, peptone was combined with corn steep powder to further improve DPEase production. As shown in Fig. 3, the highest DPEase activity (246.4 ± 6.6 U/mL) was obtained when the concentrations of peptone and corn steep powder were 20 g/L and 15 g/L, respectively.

**Table 1:** The effect of different nitrogen sources on cell growth (DCW) and recombinant DPEase activity in shake flasks

Nitrogen source	Concentration (g/L)	DCW (g/L)	DPEase activity (U/mL)
Yeast extract	15	4.1 ± 0.1	137.6 ± 2.8
	25	5.2 ± 0.2	162.0 ± 3.4
	35	5.3 ± 0.1	127.7 ± 3.2
Peptone	15	4.1 ± 0.1	164.1 ± 3.5
	25	5.1 ± 0.1	189.9 ± 2.8
	35	5.4 ± 0.2	186.4 ± 2.6
Yeast decoction	15	3.2 ± 0.1	122.4 ± 3.9
	25	3.4 ± 0.1	128.4 ± 2.9
	35	4.0 ± 0.1	142.6 ± 3.5
Corn steep powder	5	2.6 ± 0.1	92.4 ± 1.9
	15	4.3 ± 0.1	167.0 ± 3.3
	25	4.4 ± 0.1	111.1 ± 2.1
	35	5.7 ± 0.2	109.0 ± 2.9
Cottonseed meal	15	3.2 ± 0.1	123.8 ± 2.7
	25	4.7 ± 0.2	199.4 ± 3.9
	35	6.2 ± 0.2	213.7 ± 4.8

#### Carbon sources and carbon source concentrations

**Fig. 4:**
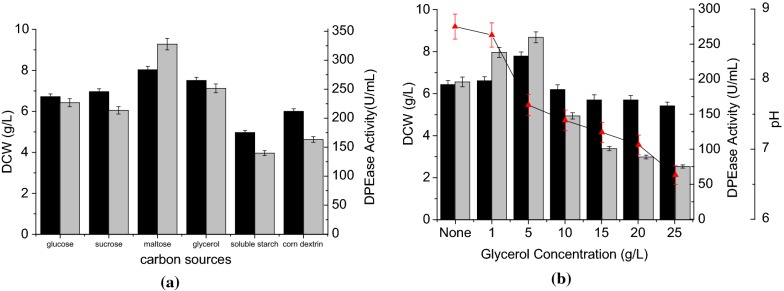
Effects of different carbon sources and carbon source concentrations on cell growth and DPEase activity. **a** The effects of 0.5% concentrations of different carbons sources; **b** the effects of different concentrations of glycerol on cell growth, DPEase production, and pH. DCW (black), DPEase activity (gray), pH (red). Error bars represent the standard deviation of measurements performed in triplicate

The identity and concentration of the carbon source are important parameters for cell growth and enzyme expression in *B. subtilis*. Thus, different carbon sources may have a significant impact on recombinant DPEase expression in *B. subtilis*. Using the combination of 20 g/L peptone and 15 g/L corn steep powder as nitrogen source, the effects of different carbon sources (0.5% glycerol, glucose, sucrose, corn dextrin and soluble starch) on cell growth and enzyme production were tested, respectively. Figure 4a displays the DCW and DPEase production profiles in the presence of different carbon sources. Cells cultured with maltose exhibited the highest biomass and DPEase production, followed by that with glycerol. However, due to the high price of maltose, we choose glycerol as the carbon source for subsequent experiments.

To determine the optimal glycerol concentration for DPEase production, glycerol with concentration varying from 1 to 25 g/L was added to the culture medium, respectively. As shown in Fig. 4b, the highest DPEase activity (259.7 ± 7.8 U/mL) was obtained with 5 g/L glycerol. When the glycerol concentration was increased, the DCW and enzyme activity decreased significantly, and the final pH in the fermentation medium also declined. These changes may result from catabolite metabolic repression (CCR), which has been noted during *B. subtilis* fermentation [27]. In CCR, the presence of an abundant carbon source has adverse effects on cell growth and recombinant protein production. Catabolite control protein A (CcpA) is the primary regulatory protein that mediates the CCR effect. CcpA binds to specific DNA sequences (CRE boxes) and inhibits gene transcription, thereby repressing protein production [28]. The expression vector used in this study contains a CRE sequence upstream of the P*amy*E promoter. The presence of the CRE sequence may cause an increase in carbon source concentration to significantly decrease DPEase expression. The CCR effect also alters metabolic pathways and can lead to the accumulation of acetic and lactic acids. Thus, the CCR effect would be expected to decrease the pH of the fermentation medium with increasing carbon concentration, which is exactly what is observed (Fig. 4b).

#### Metal ions

**Fig. 5:**
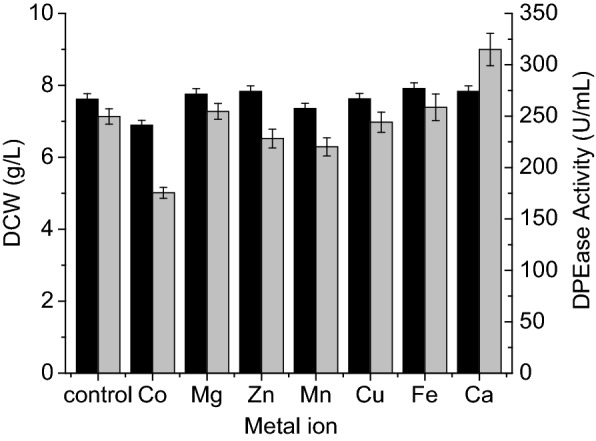
Effects of trace metal ions on cell growth and DPEase activity. DCW (black), DPEase activity (gray). All metal ions were tested at 1 mM concentration. Error bars represent the standard deviation of measurements performed in triplicate

To investigate the effects of trace metal ions on the production of recombinant DPEase in *B. subtilis*, different metal ions were added to the fermentation medium at a final concentration of 1 mM, respectively. As seen in Fig. 5, the addition of most metal ions had no effect on cell growth or enzyme production. Some metal ions, including Co^2+^ and Mn^2+^, inhibited DPEase production to varying extents. Only Ca^2+^ increased DPEase production, resulting in a DPEase activity of 314 U/mL, which was 26% higher than that of the control.

### The effects of different ratios of feeding carbon source to nitrogen source on the production of DPEase in 3.6-L fermenter

The fed-batch cultivation strategy is often used to obtain high cell density and protein expression. When implementing this strategy, several parameters (medium component and concentration, temperature, pH, dissolved oxygen, et al.) must be optimized to increase target protein production [29–31]. The shake-flask experiments described above confirmed that the carbon source concentration has a substantial influence on cell growth and DPEase production and that elevated carbons source levels lead to a CCR effect. Strategies to minimize the CCR effect in *B. subtilis* have included adjusting the glucose flow rate while monitoring the pH and carbon concentration [32] and maintaining the glucose concentration at a low level [33]. In our previous work, when we maintained the carbon source concentration below 1 g/L during fed-batch fermentation in a 3.6-L fermenter, the yield of recombinant DPEase in *B. subtilis* was only 503 U/mL (1.7 g/L). We also tried controlling the feeding rate during different growth stages as well as adjusting the fermentation temperature, pH, dissolved oxygen content and other parameters. However, the results were still not ideal. As an alternative to previous approaches, we decided to manipulate the ratio of carbon to nitrogen (C/N) while controlling the feeding medium concentration at 500 g/L to ensure the needs of nutrition. To test this approach, fed-batch experiments were used to assess the effects of different C/N ratios on DPEase production.

**Fig. 6:**
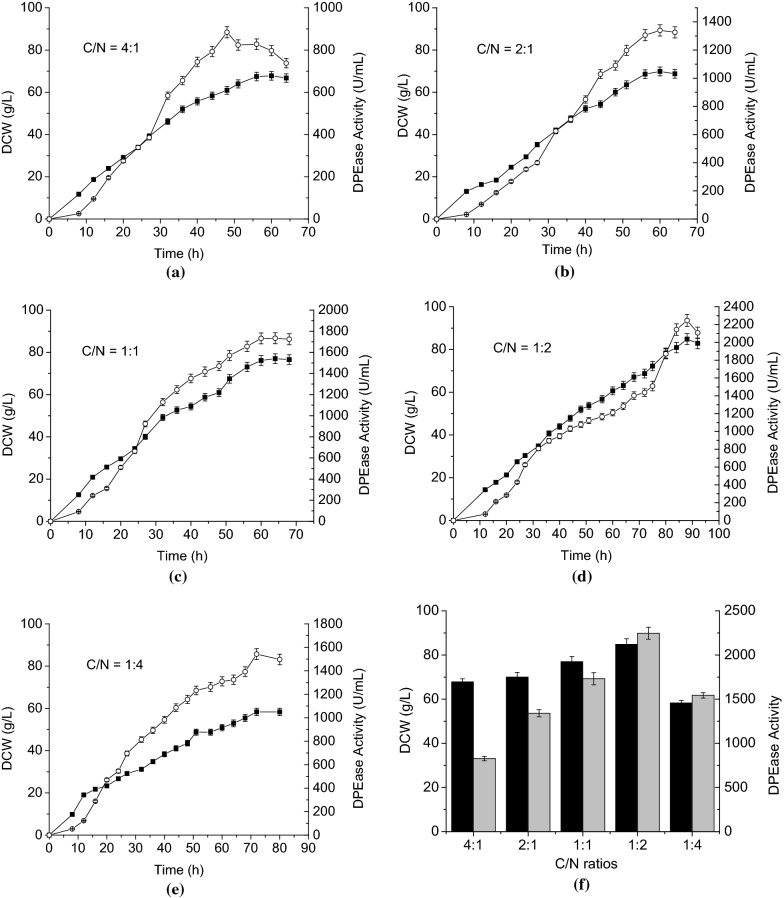
Effects of different carbon source to nitrogen source (C/N) ratios in feeding solution on cell growth and DPEase activity obtained during fed-batch fermentation in a 3.6-L fermenter. **a**–**e** DCW (filled square), DPEase activity (empty circle). **f** DCW (black), DPEase activity (gray). Error bars represent the standard deviation of measurements performed in triplicate

The results showed that the varied C/N ratio in the feeding medium had an effect on cell growth and DPEase production (Fig. 6). When the concentration of nitrogen in the feeding medium was higher, cell growth was slower and the fermentation period was longer. At a C/N ratio of 4:1, the DCW reached its peak at about 56 h, while at a C/N ratio of 1:2, the DCW reached its peak at 88 h (Fig. 6a–d). When the slow-acting nitrogen source peptone was used, its rate of absorption and utilization was not as fast as that of the fast-acting carbon source glycerol, resulting in a longer fermentation period. It can also be seen (Fig. 6a–d) that when C/N ratio was varied from 4:1 to 1:2, DPEase activity and cell growth increased with increasing nitrogen concentration. The highest DPEase activity (2246 U/mL) was obtained at 88 h when C/N ratio was 1:2. When the carbon concentration was increased, the DPEase activity decreased significantly. The cell density at C/N ratio of 1:2 was 1.25 times greater than that observed at C/N ratio of 4:1 and the DPEase activity was 2.71 times greater. These results likely reflect the CCR effect; elevated carbon source concentrations inhibited DPEase gene transcription and, therefore, protein production. The CCR effect could be effectively alleviated by adjusting the C/N ratio in the feeding medium to 1:2. When the C/N ratio was further lowered to 1:4 (Fig. 6e), the DCW reached its peak (58 g/L) at 72 h and declined much earlier than was seen at C/N ratio of 1:2. The DPEase activity was also substantially lower at C/N ratio of 1:4 than that at C/N ratio of 1:2. Apparently, shifting the C/N ratio too far toward the nitrogen source is not conducive to bacterial growth and recombinant protein production; there is a lower limit below which the carbon source concentration limits cell growth and recombinant protein production.

The effects of different C/N ratios on cell growth and DPEase activity in fed-batch fermentation are summarized in Fig. 6f. DCW (85 g/L) and DPEase activity (2246 U/mL; 7.8 g/L) were highest with 1:2 ratio of C/N, which was the optimal condition for DPEase production via high-density fermentation. This DPEase activity level was 15 times higher than that seen in shake-flask fermentations and 4.5 times higher than that obtained by maintaining the carbon source concentration below 1 g/L using conventional fed-batch fermentation techniques. The final recombinant DPEase production level was also threefold higher than the highest level reported to date.

To the best of our knowledge, this is the first report to enhance recombinant DPEase production in a food-grade expression system using an efficient and convenient fermentation strategy. In this study, the feeding rate (6 g/L/h) remained constant during the fermentation period, eliminating the need to adjust the feeding rate to maintain a low carbon source concentration. Thus, we could obtain high protein production using a process that is easy to control and conducive to industrial applications.

### Bioconversion of d-fructose to d-psicose by recombinant *B. subtilis* cells

DPEase is an intracellular enzyme. To obtain substantial quantities during industrial production, cells must be lysed and the recombinant enzyme purified, which is both time-consuming and expensive. The substrate (d-fructose) and product (d-psicose) of DPEase are small molecules that easily enter and exit cells. Bioconversion of d-fructose to d-psicose by whole-cell catalysis has the potential to avoid DPEase extraction and purification, improve enzyme stability, and meet the needs of industrial d-psicose production. Therefore, we assessed the use of our DPEase expression system to produce d-psicose directly. The effects of wet cell dosage, temperature and pH on d-psicose production were investigated.

**Fig. 7:**
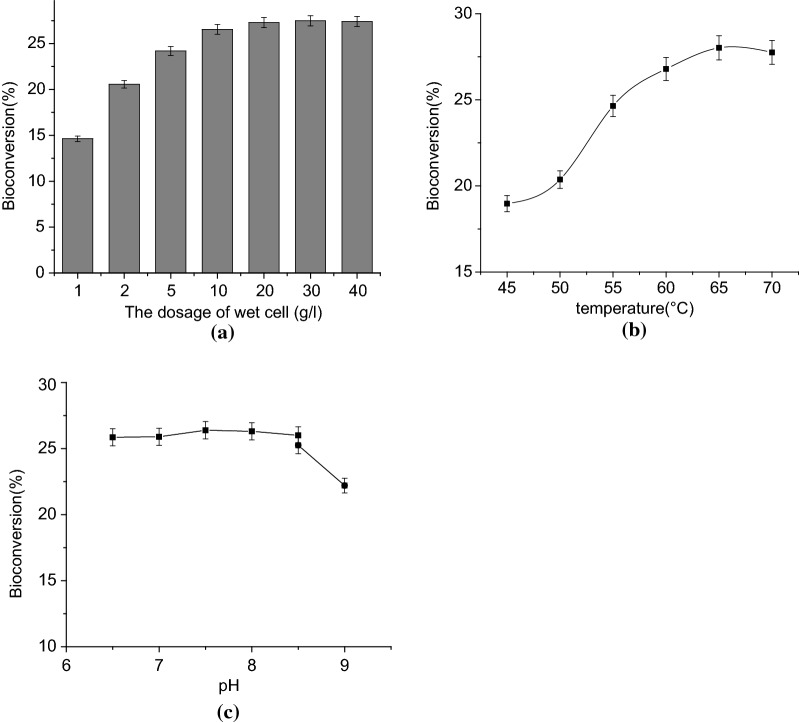
Effects of wet cell dosage (**a**), temperature (**b**) and pH (**c**) on d-psicose production by *B. subtilis* CCTCC M 2016536 containing the DPEase expression plasmid pHY300PLK-DPEase. Error bars represent the standard deviation of measurements performed in triplicate

The optimal cell concentration for d-psicose production was determined by varying the wet cell dosage from 1 g/L to 40 g/L. d-Psicose production increased with increasing cell concentration, essentially reaching a plateau at 10 g/L (Fig. 7a). Therefore, a wet cell concentration of 10 g/L was used during subsequent experiments. The turnover yield was then examined at temperatures ranging from 45 to 70 °C (Fig. 7b). Maximal d-psicose production (about 28% conversion) was obtained at 65 °C. Production decreased below 65 °C, perhaps because high temperature helped increase cell permeability. Fortunately, higher temperatures are preferred in industrial applications because they reduce the chances of contamination. The effect of pH on d-psicose production was then investigated at 65 °C and 10 g/L wet cell by varying pH from 6.5 to 9.0. The result showed that optimal conversion of d-fructose to d-psicose could be achieved over a wide pH range, from 6.5 to 8.5 (Fig. 7c). This broad pH optimum is also advantageous for industrial applications.

## Conclusions

The DPEase from *C. cellulolyticum* H10 was expressed in *Bacillus subtilis*, and fermentation conditions were optimized to improve DPEase production. A feeding strategy that combined a low carbon substrate concentration with a constant feeding rate in a 3.6-L fermenter was proposed. The resulting DPEase expression level (2246 U/mL; 7.8 g/L) achieved was three times greater than the highest level reported to date. The recombinant *B. subtilis* cells were further used in synthesis of d-psicose, and the conversion rate of 28% was achieved. This study provided a simple and convenient strategy to minimize the CCR effect in recombinant *B. subtilis* fermentation and established a basis for industrial scale production and application of DPEase.
